# Structure and Measurement Invariance of Ethnic Identity for Native American College Students

**DOI:** 10.3389/fpsyg.2019.01651

**Published:** 2019-07-26

**Authors:** Li Lin, Dexin Shi, Lori Anderson Snyder, Taehun Lee, William Demar Taylor

**Affiliations:** ^1^Human Resources Center of Excellence, PepsiCo, Inc., Purchase, NY, United States; ^2^Department of Psychology, University of South Carolina, Columbia, SC, United States; ^3^Department of Psychology, The University of Oklahoma, Norman, OK, United States; ^4^Department of Psychology, Chung-Ang University, Seoul, South Korea; ^5^Talent Assessment and Analytics Program, Human Resources Research Organization, Alexandria, VA, United States

**Keywords:** ethnic identity, Native American, Multigroup Ethnic Identity Measure, bifactor structure, multi-group analysis

## Abstract

With a specific focus on the Native American population, the current study investigated the structure of ethnic identity, measured by the Multigroup Ethnic Identity Measure, using a bifactor model across Native American (*n* = 307), Asian (*n* = 348), and White (*n* = 549) undergraduate students. We further investigated measurement invariance across ethnic groups that shared the same factor structure. The results indicated that ethnic identity can be modeled by a bifactor structure with a general factor and two group factors, affective pride and exploration, for Native American and Asian respondents but not White respondents. In addition, measurement invariance tests supported partial weak invariance between the Native American group and the Asian group. The current findings suggest that comparisons of ethnic identity scores across ethnic groups should be treated with caution.

## Introduction

Social identity, which stems from a person’s recognition of and emotional attachments to a particular social group, is an important part of a person’s self-concept (Tajfel, 1981). Specific to ethnicity or ethnic group membership, ethnic identity has been defined as a person’s recognition, feelings, and attachment to an ethnic group (Phinney, 1992). Research has supported the notion that ethnic identity is a critical component of a person’s self-evaluation. Particularly, ethnic identity is related to self-esteem, self-acceptance, and psychological adjustment (Smith and Silva, 2011). Moreover, the construct plays a significant role in the psychological well-being of ethnic minority individuals (Phinney, 1992; Kenyon and Carter, 2011).

Compared with other ethnic groups, Native Americans have unique historical and contemporary experiences, which may further complicate their perceptions of group membership and identification (Yetter and Foutch, 2013). However, Native Americans are seldom included in quantitative studies of ethnic identity. As a result, unique aspects of the conception of ethnic identity to Native Americans are left unknown. Moreover, in primary studies where samples may consist of multiple ethnic groups, it is critical to understand whether ethnic identity can be compared across different ethnic groups. For example, can Native Americans’ ethnic identity be quantitatively compared with that of another group?

According to the United States Census Bureau (2012), ethnic minority groups will represent 57% of the total population in 2060, and no group will make up a majority by 2043. The assessment, evaluation, and comparison of ethnic identity are becoming more indispensable as the society increases in diversity. Through exploring the psychometric properties of the Multigroup Ethnic Identity Measure (MEIM; Phinney, 1992), the current study aimed to further understand the meaning of ethnic identity as a construct and whether the ethnic identity of Native Americans is different from those of other groups.

Phinney’s (1992) original ethnic identity research supported a one-factor model for the MEIM. However, an analysis conducted by Roberts et al. (1999) supported a two-factor structure using a 12-item scale (Supplementary Figure 1). In Roberts et al.’s (1999) two-factor solution, the first factor was labeled affirmation, belonging, and commitment. The second factor was labeled exploration. Although researchers (e.g., Lee and Yoo, 2004; Gaines et al., 2010) have proposed the possibility of a third factor, reflecting the actions that one takes to learn about and participate in ethnic-related activities, this factor has a considerable amount of overlap with the existing factors. From a theoretical standpoint, scholars (e.g., Phinney and Ong, 2007) suggested that actions and behaviors are subject to environmental constraints, such as the lack of resources or access to materials that are needed to engage in ethnic-related events, and therefore, should not be defined as a component of the construct.

Researchers (e.g., Yap et al., 2014) have also suggested that ethnic identity, measured by the MEIM, may be better understood using a bifactor structure. A bifactor structure is a hierarchy of a general factor and subfactors (i.e., group factors). Specifically, the general factor explains the covariance among all items while the orthogonal group factor(s) capture the remaining covariance among subsets of items (Chen et al., 2006; Reise et al., 2007). The bifactor structure allows researchers to distinguish between the primary dimension of interest and several secondary dimensions. For example, in achievement tests, the primary dimension of interest captures the targeted process skills while the subfactors describe domain-specific knowledge (Gibbons et al., 2009).

Drawing from the developmental model of ethnic identity achievement, which views ethnic identity as a developmental process that follows the stages of exploration and commitment (Marcia, 1966), it is possible that ethnic identity can be best represented with a bifactor structure which consists of an overarching factor and subfactors that represent exploration and commitment. Alternatively, previous studies (e.g., Lee and Yoo, 2004) have suggested that there seems to be a cognitive component of the construct, which represents the understanding and recognition of one’s ethnic identity. It is possible that the cognitive component comprises most of the variance in ethnic identity. In this hypothetical structure, one would develop awareness of one’s ethnic identity and its meaning to him or her, while continuing to explore one’s ethnic identity and develop emotions, specifically affective pride, toward his or her ethnic group. In such a case, the items that represent the cognitive component of ethnic identity would be loading onto the overarching factor rather than forming a subfactor, while items that represent affective pride and exploration would be loading onto two subfactors. In either bifactor structure scenario, one primary dimension (the overarching factor of ethnic identity) and secondary dimensions can be identified. Consequently, we hypothesized the following:

**Hypothesis:** Ethnic identity can be modeled using a bifactor structure, which consists of a general factor, reflecting the primary dimension of interest (e.g., understanding of one’s ethnic identity), and subfactors, reflecting secondary dimensions (e.g., affective pride and exploration behavior toward one’s ethnic identity).

### Measurement Invariance of the Multigroup Ethnic Identity Measure

The MEIM was originally developed as a tool that can assess ethnic identity across people regardless of their ethnic group (Phinney, 1992). However, it is unclear whether ethnic identity carries the same meaning across people of different ethnic groups. In other words, it is not clear whether the factor structure of the construct is invariant across different ethnic groups. Several studies (e.g., Avery et al., 2007; Gaines et al., 2010) found evidence that supports configural invariance but not metric invariance. That is, the construct was structurally similar across groups, but the loadings from items to their respective proposed factors were not. However, using a bifactor model structure, Yap et al. (2014) found support for metric invariance for ethnic identity across several ethnic groups. The mixed findings indicate that further investigation is needed to understand the psychometric properties of ethnic identity across different ethnic groups.

Previous quantitative research on the ethnic identity of Native Americans as compared with those of other ethnic groups has been lacking. The ethnic identity of Native Americans is unique in its heterogeneity in terms of ancestry, tribal enrollment policies, and cultural affiliations (Hawkins et al., 2004). Historic events such as relocation and assimilation may be particularly impactful to Native Americans’ ethnic identity, and thus, the psychometric properties of ethnic identity may be different. The current study sought to further investigate the invariance properties of ethnic identity, particularly, whether ethnic identity for Native Americans is different from that of other groups.

**Research Question:** Will the general factor that represents ethnic identity be invariant between Native Americans and other ethnic group(s)?

## Methods

### Participants and Procedures

Participants consisted of 1,204 undergraduate students (61% female and 39% male) from a large research university in the southcentral region of the United States. Three self-reported ethnic groups were included in the analysis: Asian/Asian American (*n* = 348), Native American (*n* = 307), and White/White American (*n* = 549). Other ethnic groups and non-U.S. Citizens were not recruited during the data collection process and consequently were not included in the current analysis. About 7% of the sample were married. About 80% of the sample have at least one parent who went to college. Students were enrolled in a vast array of majors, including humanities, business, journalism, chemistry, biology, and engineering.

Data used in the current study originated from a large-scale, longitudinal study that investigates Native Americans’ interest and participation in science and engineering fields. Participants were invited to complete an online survey, which took about 40 min on average. Participants were compensated with a gift card for the completion of the survey.

### Measure

#### Ethnic Identity

Participants’ ethnic identity was measured by Roberts et al.’s (1999) 12-item version of the MEIM (Supplementary Figure 1). Participants indicated the ethnic group that they primarily identify with and then rated the items using a scale from 1 (strongly disagree) to 4 (strongly agree). The Cronbach’s alphas for Native American, Asian, and White students were 0.92, 0.91, and 0.86 respectively.

### Analysis

#### Model Comparison

To test our hypothesis, we fitted two bifactor models using the robust maximum likelihood (RML) estimation method to the White, Native American, and Asian groups separately. The first bifactor model, found in Yap et al. (2014), is referred to as Bifactor (E+C) in the current study (Supplementary Figure 2). The model consisted of two group factors, Exploration (Items 1, 2, 4, 8, and 10) and Commitment (Items 3, 5, 6, 7, 9, 11, and 12).

As discussed previously, it is possible that the cognitive component (Items 3, 6, and 7) of the MEIM should load onto the general factor (ethnic identity) only. That is, one’s recognition, understanding, and thoughts about his or her ethnic group is the general ethnic identity construct rather than a subcomponent of it. Thus, we tested a second bifactor model (Supplementary Figure 3) with a general ethnic identity factor and two group factors, Affective Pride and Exploration (i.e., P + E). This model is referred to as Bifactor (P+E) in the current study. All current models were conducted in Mplus version 7.11 (Muthén and Muthén, 1998-2012). We utilized common fit indices and applied common cut-off criteria that researchers (e.g., Schermelleh-Engel et al., 2003) have recommended to evaluate the models.

#### Measurement Invariance

To determine whether the general factor in the selected bifactor model is invariant across ethnic groups, we followed recommendations in the literature (e.g., Vandenberg and Lance, 2000) to test configural invariance (i.e., equivalence of structure), weak (metric) invariance (i.e., equivalence of factor loadings), and strong invariance (i.e., equivalence of intercepts and factor loadings). We applied the Satorra-Bentler (SB) scaled chi-square difference tests (Satorra and Bentler, 2001) to determine whether the model with more constraints fits the data equally well as the model with fewer constraints (see Cheung and Rensvold, 2002; Millsap, 2012 regarding measurement invariance techniques).

## Results

### Results of Model Comparison

The descriptive statistics and inter-item correlations for each group are reported in Table 1. The fit indices for all models are reported in Table 2. Based on the common fit indices, the two bifactor models were similar in terms of model fit.

**TABLE 1:** Correlations and descriptive statistics by groups.

**Group**	**Item**	***M***	***SD***	**1**	**2**	**3**	**4**	**5**	**6**	**7**	**8**	**9**	**10**	**11**	**12**
White *n* = 549	1	2.23	0.92	1.00											
	2	2.30	0.91	0.22	1.00										
	3	2.54	0.84	0.28	0.28	1.00									
	4	2.11	0.89	0.40	0.29	0.37	1.00								
	5	3.11	0.66	0.12	0.16	0.26	(0.09)	1.00							
	6	2.66	0.84	0.20	0.29	0.48	0.27	0.53	1.00						
	7	2.68	0.80	0.19	0.30	0.56	0.33	0.36	0.61	1.00					
	8	2.08	0.85	0.60	0.27	0.38	0.47	0.12	0.26	0.33	1.00				
	9	2.56	0.88	0.28	0.16	0.40	0.22	0.42	0.46	0.42	0.32	1.00			
	10	2.30	0.91	0.31	0.32	0.37	0.30	0.19	0.42	0.41	0.41	0.35	1.00		
	11	2.40	0.85	0.29	0.29	0.47	0.30	0.40	0.63	0.51	0.41	0.60	0.56	1.00	
	12	2.87	0.72	0.17	0.19	0.31	(0.10)	0.51	0.40	0.29	0.20	0.57	0.27	0.51	1.00
Native American *n* = 307	1	3.10	0.79	1.00											
	2	2.39	0.93	0.47	1.00										
	3	2.87	0.83	0.56	0.51	1.00									
	4	2.62	0.87	0.47	0.40	0.43	1.00								
	5	3.51	0.59	0.43	0.27	0.41	0.31	1.00							
	6	2.85	0.85	0.43	0.43	0.65	0.34	0.47	1.00						
	7	2.97	0.81	0.49	0.37	0.74	0.39	0.43	0.74	1.00					
	8	2.88	0.90	0.67	0.46	0.62	0.44	0.44	0.55	0.63	1.00				
	9	3.30	0.71	0.53	0.34	0.50	0.37	0.60	0.49	0.53	0.55	1.00			
	10	2.64	0.99	0.55	0.63	0.65	0.41	0.35	0.57	0.58	0.63	0.53	1.00		
	11	2.91	0.90	0.62	0.48	0.70	0.45	0.48	0.69	0.69	0.65	0.64	0.73	1.00	
	12	3.36	0.63	0.43	0.27	0.47	0.26	0.50	0.52	0.45	0.42	0.59	0.45	0.58	1.00
Asian *n* = 348	1	2.78	0.84	1.00											
	2	2.59	1.00	0.32	1.00										
	3	3.08	0.75	0.34	0.42	1.00									
	4	2.86	0.88	0.41	0.44	0.40	1.00								
	5	3.21	0.68	0.40	0.36	0.43	0.40	1.00							
	6	2.84	0.85	0.35	0.47	0.53	0.41	0.58	1.00						
	7	2.97	0.76	0.38	0.51	0.66	0.42	0.58	0.72	1.00					
	8	2.80	0.86	0.53	0.48	0.45	0.43	0.42	0.52	0.47	1.00				
	9	3.05	0.79	0.41	0.35	0.49	0.38	0.57	0.52	0.49	0.55	1.00			
	10	3.04	0.80	0.39	0.44	0.51	0.29	0.44	0.59	0.56	0.46	0.48	1.00		
	11	2.88	0.82	0.40	0.40	0.46	0.36	0.53	0.68	0.52	0.50	0.62	0.60	1.00	
	12	3.18	0.67	0.35	0.30	0.48	0.27	0.62	0.59	0.53	0.46	0.68	0.51	0.61	1.00

*All coefficients are significant at *p* < 0.01. Those in parenthesis are significant at *p* < 0.05.*

**TABLE 2:** Fit indices across models.

**Group**	**Model**	**AIC**	**BIC**	**χ^2^ (*df*)**	**CFI**	**TLI**	**RMSEA**	**SRMR**
White	Bifactor (E+C)	14046.011	14252.800	166.973 (42)	0.937	0.901	0.074 [0.062, 0.086]	0.041
	Bifactor (P+E)	14055.099	14248.963	177.589 (45)	0.933	0.902	0.073 [0.062, 0.085]	0.044
Native American	Bifactor (E+C)	6792.666	6971.555	128.534 (42)	0.954	0.928	0.082 [0.066, 0.098]	0.035
	Bifactor (P+E)	6805.004	6972.712	145.225 (45)	0.947	0.922	0.085 [0.070, 0.101]	0.039
Asian	Bifactor (E+C)^*^	7993.027	8177.933	116.294 (42)	0.954	0.928	0.071 [0.056, 0.087]	0.037
	Bifactor (P+E)	8011.686	8185.035	132.661 (45)	0.946	0.921	0.075 [0.060, 0.090]	0.039

All chi-square values are significant. ^*^ indicates model with inadmissible solution (i.e., negative residual variance). AIC = Akaike Information Criterion, BIC = Bayesian Information Criterion, CFI = Comparative Fit Index, TLI = Tucker Lewis Index, RMSEA = Root Mean Square Error Of Approximation; the corresponding 95% confident interval for RMSEA is reported in the bracket. SRMR = Standardized Root Mean Square Residual, Bifactor (E+C) = Bifactor model with Commitment and Exploration as group factors, Bifactor (P+E) = Bifactor model with Affective Pride and Exploration as group factors.

However, as shown in Table 3, Bifactor (E+C) contains several non-significant loadings (Items 3 and 7 for White, Item 11 for Native American, and Items 5 and 10 for Asian students). In addition, similar to results found in Yap et al. (2014), for both the Native American and Asian groups, Items 3, 6, and 7 loaded negatively to the group factor, Commitment. This would imply that the higher the score on Items 3, 6, and 7, the lower the Commitment, which is illogical. Moreover, the estimate of residual variance for item 7 was negative for the Asian group. Thus, Bifactor (E+C) should not be viewed as the most appropriate model for the current data.

**TABLE 3:** Standardized factor loadings for the bifactor models.

**Items**	**White**				**Native American**				**Asian**			
	**Bifactor (E+C)**		**Bifactor (P+E)**		**Bifactor (E+C)**		**Bifactor (P+E)**		Bifactor (E+C)^*^		**Bifactor (P+E)**	
	**General**	**Group**	**General**	**Group**	**General**	**Group**	**General**	**Group**	**General**	**Group**	**General**	**Group**
3	0.669	(0.028)	0.655	–	0.819	−0.222	0.833	–	0.664	−0.194	0.701	–
6	0.752	0.181	0.786	–	0.790	−0.15	0.790	–	0.806	−0.103	0.843	–
7	0.764	(−0.009)	0.745	–	0.830	−0.228	0.820	–	0.814	−0.630	0.828	–
5	0.431	0.455	0.486	0.364	0.595	0.322	0.527	0.490	0.721	(0.002)	0.671	0.300
9	0.559	0.470	0.603	0.385	0.735	0.453	0.638	0.493	0.759	0.195	0.655	0.465
11	0.743	0.299	0.774	0.207	0.870	(0.016)	0.849	0.168	0.786	0.194	0.732	0.238
12	0.394	0.733	0.454	0.780	0.642	0.274	0.542	0.495	0.774	0.150	0.675	0.515
1	0.323	0.662	0.321	0.654	0.668	0.267	0.561	0.548	0.491	0.448	0.480	0.485
2	0.400	0.154	0.389	0.167	0.515	0.571	0.482	0.300	0.533	0.296	0.577	0.222
4	0.428	0.397	0.406	0.414	0.492	0.256	0.500	0.291	0.479	0.351	0.503	0.313
8	0.465	0.677	0.451	0.691	0.748	0.216	0.716	0.376	0.626	0.462	0.628	0.458
10	0.603	0.176	0.595	0.190	0.742	0.371	0.746	0.183	0.692	(0.072)	0.711	(0.038)

Coefficients in the parenthesis are not significant. All other parameters are significant. ^*^ indicates model with inadmissible solution (i.e., negative residual variance for item 7). Bifactor (E+C) = Bifactor model with Commitment and Exploration as group factors, Bifactor (P+E) = Bifactor model with Affective Pride and Exploration as group factors. The “General” column indicates factor loadings on the general factor. The “Group” column indicates the factor loadings on the corresponding group factors.

On the other hand, the model results for Bifactor (P+E) were more interpretable (Table 3). Unlike Bifactor (E+C), all items in Bifactor (P+E) had higher loadings on the general factor than on the group factors for the Native American group, implying that most of the item variance was explained by the overarching factor. The same pattern holds for the Asian group with the exception of Item 1. In comparison, four items (Items 1, 4, 8, and 12) in the White group had smaller loadings on the general factor than on the group factors.

Based on results of Bifactor (P+E), to further examine whether a primary dimension of interest is present in ethnic identity, we adopted Rodriguez et al.’s (2016) suggestion, and computed the proportion of common variance across items that was explained by the general factor and each of the group factors. For the Native American group, the general factor, the Affective Pride group factor, and the Exploration group factor each accounted for 82.5, 7.2, and 10.3% of the items’ common variance, respectively. For the Asian group, the corresponding percentages of variance accounted for were 82.0, 7.7, and 10.3%, respectively. For the White group, the general factor only accounted for 66.9% of the common variance, which is notably lower in comparison with the other two groups; the group factors accounted for the remaining 33.1% of the common variance (11.9% for Affective Pride and 21.2% for Exploration).

Thus, we conclude that our hypothesis was partially supported, such that a general factor of ethnic identity emerged in the bifactor model, Bifactor (P+E), for the Native American group and the Asian group. However, the evidence for the presence of this general factor was not clear for the White group. Given this, the tests of measurement invariance using Bifactor (P+E) would only be appropriate between the Native American group and the Asian group.

### Results of Measurement Invariance Tests Between Native American and Asian

The current research question asks whether ethnic identity can be regarded as invariant between Native American and another ethnic group. To answer this question, we selected Asians as the comparison group. In the tests of measurement invariance, the baseline model with the fewest constraints (reported in Table 4) provided a reasonable fit to the data [χ^2^(90) = 284.338, CFI = 0.947, TLI = 0.922, RMSEA = 0.077, SRMR = 0.039], granting support for configural invariance. However, the weak invariance assumption [χ^2^(101) = 336.436, CFI = 0.935, TLI = 0.915, RMSEA = 0.080, SRMR = 0.072], which states that the factor loadings from the items to the general factor are equivalent across groups, did not hold [Δχ^2^(11) = 54.72, *p* < 0.001]. Provided that not all factor loadings are equivalent across the two groups, we examined which factor loadings are non-invariant by testing partial weak invariance using suggestions and guidelines found in Byrne et al. (1989) and Shi et al. (2017).

**TABLE 4:** Parameter estimates for the baseline model.

	**Asian**					**Native American**				
	**T**	**λ_GF_**	**λ_P_**	**λ_E_**	**ε**	**τ**	**λ_GF_**	**λ_P_**	**λ_E_**	**ε**
Item1^*^	2.784	0.401	0.405	–	0.373	3.100	0.480	0.405	–	0.304
Item2	2.589	0.579	0.223	–	0.622	2.384	0.451	0.881	–	0.371
Item3	3.078	0.527	–	–	0.288	2.867	0.638	–	–	0.209
Item4	2.865	0.440	0.274	–	0.497	2.619	0.392	0.410	–	0.521
Item5^*^	3.210	0.457	–	0.204	0.214	3.506	0.283	–	0.204	0.178
Item6	2.839	0.713	–	–	0.207	2.848	0.621	–	–	0.261
Item7^*^	2.968	0.626	–	–	0.180	2.968	0.626	–	–	0.194
Item8	2.796	0.537	0.391	–	0.290	2.871	0.617	0.342	–	0.311
Item9	3.055	0.513	–	0.364	0.219	3.296	0.426	–	0.312	0.137
Item10	3.043	0.571	0.031	–	0.318	2.639	0.683	0.607	–	0.304
Item11	2.882	0.598	–	0.195	0.272	2.902	0.716	–	0.093	0.192
Item12	3.175	0.453	–	0.345	0.125	3.352	0.337	–	0.195	0.196
μ_GF_	0					0.009				
μ_P_	0					0.000				
μ_E_	0					0.000				
σ^2^_GF_	1					1.184				
σ^2^_P_	1					1.626				
σ^2^_E_	1					0.314				

Parameters with ^*^ are reference indicators. Factor means were constrained to be 0 and factor variances were constrained to be 1 for the Asian group. τ = intercepts; λ_*GF*_ = factor loadings for the general factor; λ_*P*_ = factor loadings for the Affective Pride group factor; λ_*E*_ = factor loadings for the Exploration group factor; ε = residual variances; μ_*GF*_ = factor means for the general factor; μ_*P*_ = factor means for the Affective Pride group factor; μ_*E*_ = factor means for the Exploration group factor; σ^2^_*GF*_ = factor variances for the general factor; σ^2^_*P*_ = factor variances for the Affective Pride group factor; σ^2^_*E*_ = factor variances for the Exploration group factor.

The measurement invariance test indicated that partial weak invariance [χ^2^(94) = 285.410, CFI = 0.945, TLI = 0.923, RMSEA = 0.079, SRMR = 0.047] can be achieved after allowing seven factor loadings to be freely estimated across groups [Δχ^2^(4) = 7.94, *p* = 0.09, ΔCFI = 0.002]. This indicates that the relationships between a majority of the items (7 out of 12) and the general ethnic identity factor are not equivalent between the Native American group and Asian group. Thus, the implication is that one should not compare ethnic identity scores between the two groups, because, for the most part, ethnic identity carries different meanings for the two groups.

## Discussion

Ethnic identity may take on more salience to a person’s identity and psychological well-being as the person’s context becomes more culturally diverse. Although Phinney’s (1992) initial development of the MEIM lays the foundation for the understanding of ethnic identity, the structural properties of the construct require additional investigation and confirmation. The current bifactor model adds a new perspective to the literature in revealing ethnic identity as a general construct that can be best represented by the extent to which an individual is aware of and understands the meaning of his or her ethnic identity. Both affective pride and behavioral exploration are also important to the construct. However, these components can be subsumed under the cognitive component of recognition and understanding. Putting this in simple terms, one cannot feel proud of his or her ethnicity or explore additional aspects of his or her ethnicity without recognizing and understanding what ethnic identity means to him or her.

In addition, given the model results about the White group, it is likely that the meaning of ethnic identity for White individuals is fundamentally different and is not comparable to those of ethnic minority groups. As Rowe et al. (1994) pointed out, the relevance of ethnic identity to White individuals may be in the aspects of White racial consciousness, which is the awareness of being White and the social expectations, stereotypes, or privileges that are associated with being White. Ethnic identity for the White group should be investigated separately.

In the test of invariance between the Native American group and Asian group, we found that only a few (5 out of 12) items in the measure can be constrained to be the same between the two groups without significantly reducing the fit of the model. Thus, the answer to our research question is that the MEIM is mostly non-invariant, or only partially invariant, between the Native American group and the Asian group. That is, the meaning of ethnic identity is more dissimilar than similar between Native Americans and Asians. Researchers (e.g., Yoon, 2011) have suggested that the salience and meaning of ethnic identity largely depends on unique historic experiences and contemporary contextual variables. The differences in experiences and context such as nativity to the land, oppression, and stereotypes may explain the lack of full metric invariance in ethnic identity for Native Americans and Asians.

### Limitations

Several limitations should be noted in the current study. First, only three ethnic groups were available to the researchers. Specific to the measurement invariance tests, the Native American group was compared with the Asian group only. It would be interesting to see how the meaning of ethnic identity differs between Native Americans and other ethnic groups. In addition, we were unable to distinguish among specific groups within the larger categories of ethnic groups. For example, about 50–60 tribes were represented in our Native American sample. However, due to complexities with formal recognition of tribes and individuals’ tribal enrollment, reliable information regarding specific tribal affiliations and sufficient numbers of students within tribal groups were unavailable.

## Future Directions and Conclusion

Future studies should also consider comparing other ethnic groups to the Native American group. It is possible that ethnic groups with similar social stereotypes would interpret ethnic identity more similarly. In general, the amount of research regarding Native Americans has been scarce. Given the focus of the current study, we referred to Native Americans as one ethnic group. Others may prefer to explore tribal identities. Despite the obfuscated distinction between racial identity and ethnic identity, with the former being more concerned with genotypical features (e.g., facial structure) and the latter more concerned with history and culture (Cokley, 2007), a rigorous attempt to interpret the two perspectives for Native Americans can provide more nuanced understanding regarding the variations and fluidity in Native American identities.

In sum, as noted by Chen (2008), non-invariant scales may lead to pseudo group differences, reflecting variations across groups in the psychometric properties of the scale rather than “real” differences. The findings of the current study suggest that comparing ethnic identity scores across White, Asian, and Native American individuals should be made with caution, and preferably avoided. Although measuring and comparing ethnic identity within one ethnic group may be reasonable, quantitative assessments of identity may be presenting an abridged version of the construct nevertheless. Scholars should also recognize that narratives that are unique to individuals are sometimes more powerful in unfolding abstract concepts.

## Data Availability

The datasets for this manuscript are not publicly available as required by the Institutional Review Board that approved the study. The current datasets were not used in other published studies. Requests to access the datasets should be directed to Dr. Lori Anderson Snyder, lsnyder@ou.edu.

## Ethics Statement

This study was carried out in accordance with the recommendations of the Institutional Review Board for the Protection of Human Subjects, The University of Oklahoma, with written informed consent from all subjects. All subjects gave written informed consent in accordance with the Declaration of Helsinki. The protocol was approved by the Institutional Review Board for the Protection of Human Subjects, The University of Oklahoma.

## Author Contributions

LL, LS, and WT led the data collection process for the current study. LL and DS led the data analysis, modeling process, and wrote the initial drafts of the manuscript. TL and LS provided guidance for modeling, writing, and editing processes. All authors were involved in the revision process and developing the initial ideas for the study.

## Conflict of Interest Statement

The authors declare that the research was conducted in the absence of any commercial or financial relationships that could be construed as a potential conflict of interest.

## References

[B1] Avery D. R. Tonidandel S. Thomas K. M. Johnson C. D. Mack D. A. (2007). Assessing the multigroup ethnic identity measure for measurement equivalence across racial and ethnic groups. *Educ. Psychol. Meas.* 67 877–888.

[B2] Byrne B. M. Shavelson R. J. Muthen B. (1989). Testing for the equivalence of factor covariance and mean structures-the issue of partial measurement invariance. *Psychol. Bull.* 105 456–466.

[B3] Chen F. F. (2008). What happens if we compare chopsticks with forks? The impact of making inappropriate comparisons in cross-cultural research. *J. Pers. Soc. Psychol.* 95 1005–1018. 10.1037/a0013193 18954190

[B4] Chen F. F. West S. G. Sousa K. H. (2006). A comparison of bifactor and second-order models of quality of life. *Multivariate Behav. Res.* 41 189–225. 10.1207/s15327906mbr4102_5 26782910

[B5] Cheung G. W. Rensvold R. B. (2002). Evaluating goodness-of-fit indexes for testing measurement invariance. *Structural Equation Modeling*, 9 233–255.

[B6] Cokley K. (2007). Critical issues in the measurement of ethnic and racial identity: a referendum on the state of the field. *J. Counsel. Psychol.* 54 224–234.

[B7] Gaines S. O. Jr. Bunce D. Robertson T. Wright B. Goossens Y. Heer D. (2010). Evaluating the psychometric properties of the multigroup ethnic identity measure (MEIM) within the united kingdom. *Identity* 10 1–19.

[B8] Gibbons R. D. Rush A. J. Immekus J. C. (2009). On the psychometric validity of the domains of the PDSQ: an illustration of the bi-factor item response theory model. *J. Psychiatr. Res.* 43 401–410. 10.1016/j.jpsychires.2008.04.013 18554611

[B9] Hawkins E. H. Cummins L. H. Marlatt G. A. (2004). preventing substance abuse in american indian and alaska native youth: promising strategies for healthier communities. *Psychol. Bull.* 130 304–323. 14979774 10.1037/0033-2909.130.2.304

[B10] Kenyon D. B. Carter J. S. (2011). Ethnic identity, sense of community, and psychological well-being among northern plains american indian youth. *J. Commun. Psychol.* 39 1–9.

[B11] Lee R. M. Yoo H. C. (2004). Structure and measurement of ethnic identity for asian american college students. *J. Counsel. Psychol.* 51 263–269.

[B12] Marcia J. E. (1966). Development and validation of ego-identity status. *J. Pers. Soc. Psychol.* 3 551–558. 5939604 10.1037/h0023281

[B13] Millsap R. E. (2012). *Statistical Approaches to Measurement Invariance.* New York, NY: Routledge.

[B14] Muthén and Muthén (1998-2012). *Mplus User’s Guide.* Los Angeles, CA: Muthén & Muthén.

[B15] Phinney J. S. (1992). The multigroup ethnic identity measure: a new scale for use with diverse groups. *J. Adolesc. Res.* 7 156–176. 10.1037/pas0000606 29902048

[B16] Phinney J. S. Ong A. D. (2007). Conceptualization and measurement of ethnic identity: current status and future directions. *J. Couns. Psychol.* 54 271–281. 10.1037/0022-0167.54.3.271

[B17] Reise S. P. Morizot J. Hays R. D. (2007). The role of the bifactor model in resolving dimensionality issues in health outcomes measures. *Qual. Life Res.* 16 19–31. 17479357 10.1007/s11136-007-9183-7

[B18] Roberts R. E. Phinney J. S. Masse L. C. Chen Y. R. Roberts C. R. Romero A. (1999). The structure of ethnic identity of young adolescents from diverse ethnocultural groups. *J. Early Adolesc.* 19 301–322.

[B19] Rodriguez A. Reise S. P. Haviland M. G. (2016). Applying bifactor statistical indices in the evaluation of psychological measures. *J. Pers. Assess.* 98 223–237. 10.1080/00223891.2015.1089249 26514921

[B20] Rowe W. Bennett S. K. Atkinson D. R. (1994). White racial identity models: a critique and alternative proposal. *Couns. Psychol.* 22 129–146.

[B21] Satorra A. Bentler P. M. (2001). A scaled difference chi-square test statistic for moment structure analysis. *Psychometrika* 66 507–514. 10.1007/s11336-009-9135-y PMC2905175 20640194

[B22] Schermelleh-Engel K. Moosbrugger H. Müller H. (2003). Evaluating the fit of structural equation models: tests of significance and descriptive goodness-of-fit measures. *Methods Psychol. Res. Online* 8 23–74.

[B23] Shi D. Song H. Lewis M. D. (2017). The impact of partial factorial invariance on cross- group comparisons. *Assessment* 10.1177/1073191117711020 [Epub ahead of print]. 28598216

[B24] Smith T. B. Silva L. (2011). Ethnic identity and personal well-being of people of color: a meta-analysis. *J. Couns. Psychol.* 58 42–60. 10.1037/a0021528 21171745

[B25] Tajfel H. (1981). *Human Groups and Social Categories.* New York, NY: Cambridge University Press.

[B26] United States Census Bureau (2012). *U.S*. *Census Bureau Projections Show a Slower Growing, Older, More Diverse Nation a Half Century From Now.* Available at: https://www.census.gov/newsroom/releases/archives/population/cb12-243.html (accessed November 10, 2018).

[B27] Vandenberg R. J. Lance C. E. (2000). A review and synthesis of the measurement invariance literature: suggestions, practices, and recommendations for organizational research. *Organ. Res. Methods* 3 4–70.

[B28] Yap S. C. Y. Donnellan M. B. Schwartz S. J. Kim S. Y. Castillo L. G. Zamboanga B. L. (2014). Investigating the structure and measurement invariance of the multigroup ethnic identity measure in a multiethnic sample of college students. *J. Couns. Psychol.* 61 437–446. 10.1037/a0036253 24660693 PMC7883400

[B29] Yetter G. Foutch V. (2013). Investigation of the structural invariance of the ethnic identity scale with native american youth. *Cult. Divers. Ethnic Minor. Psychol.* 19 435–444. 10.1037/a0032564 23731231

[B30] Yoon E. (2011). Measuring ethnic identity in the ethnic identity scale and the multigroup ethnic identity measure-revised. *Cult. Divers. Ethnic Minor. Psychol.* 17 144–155. 10.1037/a0023361 21604838

