# Supplementary material for: Structure and Measurement Invariance of Ethnic Identity for Native American College Students
Source: Front Psychol. 2019 Jul 26;10:1651. doi: 10.3389/fpsyg.2019.01651 (PMC6677032; doi:10.3389/fpsyg.2019.01651)
Supplement: Supplementary file 1 [file Table_1.DOCX]

Supplementary Material

# Supplementary Figure

| Item # | Content |
| --- | --- |
| Item 1 | I have spent time trying to find out more about my ethnic group, such as its history, traditions, and customs. |
| Item 2 | I am active in organizations or social groups that include mostly members of my own ethnic group. |
| Item 3 | I have a clear sense of my ethnic background and what it means for me. |
| Item 4 | I think a lot about how my life will be affected by my ethnic group membership. |
| Item 5 | I am happy that I am a member of the group I belong to. |
| Item 6 | I have a strong sense of belonging to my own ethnic group. |
| Item 7 | I understand pretty well what my ethnic group membership means to me. |
| Item 8 | To learn more about my ethnic background, I have often talked to other people about my ethnic group. |
| Item 9 | I have a lot of pride in my ethnic group and its accomplishments. |
| Item 10 | I participate in cultural practices of my own group, such as special food, music, or customs. |
| Item 11 | I feel a strong attachment towards my own ethnic group. |
| Item 12 | I feel good about my cultural or ethnic background. |

**Supplementary Figure 1.** Multigroup Ethnic Identity Measure (MEIM)

**Item3**

**Item6**

**Item7**

**Item9**

**Item5**

**Item11**

**Item12**

**Item1**

**Item2**

**Item8**

**Item4**

**Item10**

**Supplementary Figure 2.** Bifactor (E+C)

**Item3**

**Item6**

**Item7**

**Item9**

**Item5**

**Item11**

**Item12**

**Item1**

**Item2**

**Item8**

**Item4**

**Item10**

**Supplementary Figure 3.** Bifactor (P+E)
